# 

**Published:** 1863-10

**Authors:** 


### DENTAL REGISTER

### OF THE WEST.

EDITED BY

J. TAFT AND GEO. WATT.

OCTOBER.—1863.

MONTHLY:

Three Dollars per Annum, in advance.

CINCINNATI:
J. TAFT, PUBLISHER.

J. TAFT, DENTIST, 172 RACE STREET.

